# Papain-Decorated Mucopenetrating SEDDS: A Tentative Approach to Combat Absorption Issues of Acyclovir via the Oral Route

**DOI:** 10.3390/pharmaceutics14081584

**Published:** 2022-07-29

**Authors:** Arshad Mahmood, Rabbia Haneef, Ahmad Z. Al Meslamani, Mohammad F. Bostanudin, Muhammad Sohail, Muhammad Sarfraz, Mosab Arafat

**Affiliations:** 1 College of Pharmacy, Al Ain University, Abu Dhabi Campus, Abu Dhabi P.O. Box 112612, United Arab Emirates; ahmad.almeslamani@aau.ac.ae (A.Z.A.M.); mohammad.bostanudin@aau.ac.ae (M.F.B.); 2 AAU Health and Biomedical Research Center (HBRC), Al Ain University, Abu Dhabi P.O. Box 112612, United Arab Emirates; muhammad.sarfraz@aau.ac.ae (M.S.); mosab.arafat@aau.ac.ae (M.A.); 3 Department of Pharmacy, COMSATS University Islamabad, Abbottabad Campus, Abbottabad 22060, Pakistan; rabiahanif884@gmail.com (R.H.); msmarwat@gmail.com (M.S.); 4 College of Pharmacy, Al Ain University, Al Ain P.O. Box 64141, United Arab Emirates

**Keywords:** acyclovir, SEDDS, papain, hydrophobic ion pairing, mucosal permeation

## Abstract

The aim of the current study was to enhance the oral bioavailability of Acyclovir (ACV) based on the papain-functionalized self-emulsifying drug delivery systems (SEDDS). The optimum control SEDDS formulation comprised of kolliphore (40%), transcutol (30%), propylene glycol (20%) and oleoyl chloride (10%). However, in the targeted SEDDS formulation, oleoyl chloride was replaced with oleoyl chloride-papain (OC-PAP) conjugate that was synthesized via an amide bond formation between the acyl halide groups of oleoyl chloride and the amino group of papain. Prior to adding in the SEDDS formulation, the newly synthesized conjugate was evaluated quantitatively by a Bradford assay that demonstrated 45 µg of papain contents per mg of the conjugate. Moreover, the conjugate formation was qualitatively confirmed through FTIR analysis and thin layer chromatography. ACV (a BCS class III drug) was incorporated into the SEDDS formulations after being hydrophobically ion paired with sodium deoxycholate, thereby making it lipophilic. The drug-loaded formulations were emulsified in the 0.1 M phosphate buffer (pH 6.8) and evaluated in vitro with respect to drug release and rabbit mucosal permeation studies. Both the formulations illustrated a very comparable drug release over a period of 4 h, afterwards, the OC-PAP-based formulation demonstrated a more sustaining effect. The extent of mucus diffusion evaluated via the silicon tube method demonstrated a 4.92-fold and a 1.46-fold higher penetration of the drug, a 3.21-fold and a 1.56-fold higher permeation through the rabbit intestinal mucus layer, and a 22.94-fold and a 2.27-fold higher retention of the drug over the intact mucosa of rabbit intestine, illustrated by OC-PAP-based nanoemulsions compared to the drug-free solution and controlled nanoemulsion, respectively. According to these in vitro results, papain-functionalized SEDDS is a promising approach for the oral delivery of ACV and many other drugs with oral bioavailability issues, however, in vivo studies in this respect have to be employed before making a comprehensive conclusion.

## 1. Introduction

Value-added delivery systems are continuously being explored for the conveyance of drugs into the body with the aim to treat and combat the ever-increasing challenges [1]. However, the oral route has still been the priority for even newer/modified systems as it has many perks including the highest patient compliance, cost-effectiveness, no requirements for sterility, a cascade of options to construct dosage forms and the luxury of self–administration [2]. At the same time, few challenges are faced with oral administration and to achieve optimum drug levels in the circulation the key factors are solubility characteristics of the chemical moieties and the permeability across biological membranes [3,4]. For instance, when we look at class III and class IV drugs as per the biopharmaceutical classification system (BCS), their limited permeation across the mucosal membranes is the major reason for low bioavailability. ACV belongs to BCS class III and possesses a good aqueous solubility at intestinal pH but at the same time a weak mucosal permeation potential. Due to the failure of passing through the mucosal barrier and being transported to the absorption cells in the GIT, enough drug concentration in the systemic circulation is not achieved via the oral route, resulting in sub-therapeutic effects of ACV. Accordingly, such drugs are delivered via the parenteral route that has its own limitations and drawbacks [5].

Different strategies were described in the literature to overcome the mucosal barrier for instance mucoadhesion and mucus permeation [6]. The most practical and fruitful approach for mucosal permeation is the incorporation of proteases in the drug delivery systems. Proteases are known to have a mucolytic activity and show immense stability in the internal environment of the body by being stable in the simulated gastric fluid. They disrupt the peptide bonds of the mucosal structure, ultimately cleaving the bonds, resulting in reversible disruption of the substructures, decreasing the viscosity of the mucous thereby clearing the path for drug permeation [7,8]. The commonly used proteases in drug delivery are papain, bromelain and trypsin, however, a number of studies in recent times indicated the higher permeability effects of papain compared to other counterparts [9,10,11]. Therefore, the placement of papain over the surfaces of the existing nano-particulate drug delivery systems can significantly enhance the bioavailability of the drug.

Lipid-based drug delivery systems are generally preferable over other systems as many drugs are kept in a solubilized state until the absorption site [12]. Among the lipid-based systems, SEDDS is the most promising with respect to enhancing the drug particle diffusion across the gastrointestinal tract, membrane permeability and lymphatic absorption due to the presence of surfactants, mediums and oils [13]. The only challenge that remained is the limitation of the scope of SEDDS for lipophilic drugs, however, in recent times the utilization of the hydrophobic ion-pairing technique has been successful in incorporating water-soluble drugs into lipid-based delivery systems [14].

Therefore, the current study was focused on the development of papain-functionalized SEDDS and the incorporation of ACV into the SEDDS after being conjugated with a hydrophobic surfactant. The newly developed conjugates were assessed, qualitatively as well as quantitatively, in order to make sure the efficiency of the reactions was high and the drug is able to release from the ion pair. Finally, the impact of papain immobilization was evaluated for enhanced permeation across the mucosa via different experimental setups and the success of the strategy was witnessed at the in vitro level. The combination of protease functionalization and hydrophobic ion-pair formation is a significantly new door that is opening for BCS class III drugs.

## 2. Materials and Methods

### 2.1. Materials

Acyclovir [CAS No.: 59277-89-3], Papain (from *Carica papaya*) [CAS Number: 9001-73-4], kolliphore EL [CAS No.: 61791-12-6], transcutol (Di(ethylene glycol) ethyl ether) [CAS No.: 111-90-0], propylene glycol [CAS No.: 57-55-6], polyethylene glycol 400 [CAS No.: 25322-68-3], glycerine [CAS No.: 56-81-5], oleoyl chloride [CAS No.: 112-77-6], sodium deoxy-cholate [CAS No.: 302-95-4], tetrahydrofuran [CAS No.: 109-99-9], and Bradford reagent were purchased from the Sigma-Aldrich. All other chemicals were of analytical grade.

### 2.2. Synthesis of Oleoyl Chloride-Papain (OC-PAP) Conjugate

**Figure 1:**
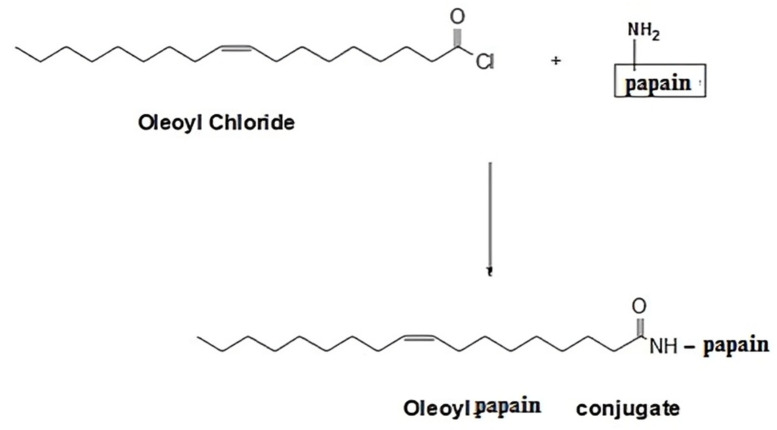
Schematic illustration of covalent linkage of papain to oleoyl chloride and hypothetical chemical formula of OC-PAP.

The enzyme conjugate was obtained by commencing out a reaction between acyl halide of oleoyl chloride and amino group of papain resulting in amide bond formation as depicted in Figure 1 below. The reaction was followed as described by Zhang and co-workers with slight modification [15]. Briefly, 1 mL of 2% (*w*/*v*) aqueous papain solution was slowly added to 10 mL of 2% (*w*/*v*) oleoyl chloride solution in tetrahydrofuran (THF) and the mixture was continuously stirred on the magnetic plate set at 40 RPM for a period of 3 h at room temperature. Afterward, the contents were transferred to a 50 mL falcon tube and an excess of water (25 to 30 mL) was added to the tube in order to create an insufficiency of solvent for the conjugate (the salting out phenomenon). The mixture in falcon tube was allowed to stand overnight at room temperature resulting in the separation of the OC-PAP as a layer above the water/THF mixture that was collected with the help of a micro-pipette.

### 2.3. Characterization of OC-PAP Conjugate

#### 2.3.1. Bradford Assay

The papain content immobilized to the oleoyl chloride was determined photo-metrically via Bradford assay [16,17]. Briefly, 1.9 mL Bradford reagent was added to 100 µL of the OC-PAP conjugate and then vortex mixing was carried out for 30 s followed by incubation at room temperature for a period of 10 mins. This solution was then transferred to the cuvette and absorbance was studied at a wavelength of 595 nm. The amount of papain in the OC-PAP conjugate was quantified via a standard curve using serial dilutions of the enzyme with a concentration range of 7.8 µg/mL–250 µg/ ml dissolved in distilled water that alone (without enzyme) acted as blank.

#### 2.3.2. Fourier Transform Infrared Spectroscopy

The confirmation of covalent linkage and formation of a new bond was appraised using FTIR analysis. For this purpose, 100 µL each of OC and OC-PAP was transferred separately to a highly polished potassium bromide solution and dried at a temperature of 45 °C. Afterward, the dry mixture was retained under a pressure of 65 kN for 5–6 s to convert the powder mixture into a 12 mm semi-transparent disk. The scan was then run over a wavelength range of 4000/cm–400/cm using a Nicolet-6700 spectrophotometer [18].

#### 2.3.3. Thin Layer Chromatography

For further confirmation of the conjugate formation, thin layer chromatography (TLC) was utilized [19]. For this purpose, pre-coated silica gel aluminum plate acted as stationary media, whereas mixture of hexane, ethyl acetate and acetic acid as the mobile phase. TLC plate of dimension 4 × 8 cm was used for the procedure. Briefly, with a pencil, a straight line was drawn at a distance of 1.5 cm from one end. A drop each of oleoyl chloride, papain solution (5 mg/mL distilled water) and OC-PAP were carefully placed on the specific points on this line. In the meantime, mobile phase was prepared by mixing hexane, ethyl acetate and acetic acid in the ratio 80:20:1, respectively. TLC plate was leveled in the mobile phase such that the line-bearing sample spots were not sunk and the mobile phase was allowed to travel through the length of plate. Later, TLC plate was taken out, dried and firstly observed under the UV lamp and then sprayed with Ninhydrin solution (66% in ethanol). After spraying, the plate was dried completely and then observed for the appearance of spots.

### 2.4. Hydrophobic Ion Pairing

Hydrophobic ion pairs were synthesized by carrying out a reaction between anionic sodium deoxy-cholate and ACV following the method described by Griesser and co-workers with some modification [14]. Briefly, the drug and the surfactant were mixed in different molar ratios of 1:2, 1:3 and 1:4, respectively. For this purpose, the respective quantities of sodium deoxy-cholate dissolved in 1 mL of distilled water against 5 mg of ACV were dissolved in 1 mL acidified (pH 3) water in Eppendorf tubes. respectively. Both the solutions, i.e., drug solution and the surfactant solution were mixed, precipitates were completely separated via centrifugation at 5000 rpm at room temperature for 10 min, these were filtered and then dried overnight at a temperature of 37 °C. In the meantime, the supernatants were collected to evaluate the remaining amount of ACV after the ion pairing, achieved via UV plate reader at the wavelength of 254 nm. A standard curve of the ACV ranging between 0–100 µg was developed for this purpose. The ion-pairing efficiency was calculated utilizing the following equation

$${\mathrm{Ion}\;\mathrm{pairing}\;\mathrm{efficiency}}^{}=100-\frac{\mathrm{Concentartion}\;\mathrm{of}\;\mathrm{ACV}\;\mathrm{after}\;\mathrm{pairing}}{\mathrm{Concentartion}\;\mathrm{of}\;\mathrm{ACV}\;\mathrm{before}\;\mathrm{pairing}}\times 100$$

### 2.5. Formulation Development of SEDDS

Initially, nine different formulations (F1–F9) of SEDDS were developed with varying amounts of components such as surfactant, co-surfactant/solvent and co-solvent one by one as described in Table 1. Each of the formulations was prepared by accurately weighing 100 mg oleoyl chloride in an Eppendorf tube to which a specified weight of kolliphore was added. The mixture was blended together via vortex mixing for 30 s, followed by the addition of respective quantities of Transcutol (solvent/co-surfactant) and one of the co-solvents. Afterward, the mixture was incubated in a thermos-mixer at 25 ± 1 °C under continuous stirring of 700 RPM for a period of 1 h. The SEDDS concentrate obtained was then stored at room temperature until further use.

### 2.6. Selection of the Stable Nanoemulsion

All the SEDDS formulations were evaluated with respect to ease of re-dispersibility and stability as nanoemulsion. For this purpose, 25 mg of each formulation was added to 10 mL of 0.1 M phosphate buffer (pH 6.8) at room temperature to form a nanoemulsion. The amount of time and shaking required to emulsify were taken as parameters for ease of emulsification. The appearance of nanoemulsion to naked eye (either transparent or translucent) at different time intervals and particle size distribution of selected formulations were taken as indicators for stability. The best-suited SEDDS formulations were selected for the drug loading and to compare the drug release pattern.

### 2.7. Drug Loading

Hydrophobically modified drug was incorporated into the selected SEDDS formulations. Briefly, the hydrophobic ion paired drug conjugate equivalent to 5 mg of the ACV was added to 500 mg of each SEDDS formulation. The mixture was vortexed until complete solubility was achieved resulting in drug-loaded SEDDS formulations. The Eppendorf tubes containing these drug-loaded SEDDS were tightly sealed and stored at room temperature for further use.

### 2.8. Drug Release Profile

Drug release profiles of selected formulations were studied via the dialysis tube method [20]. The purpose of this study was to look for the most optimized formulation in terms of sustained and complete drug release. Briefly, 50 mg of each drug-loaded SEDDS formulation (F3, F4 and F7) was emulsified in 10 mL phosphate buffer within the dialysis membrane (3 kDa) to form a nanoemulsion. The membrane was then placed in a beaker containing 25 mL phosphate buffer (pH 6.8) under sink conditions. A stirring magnet was placed in the beaker and the whole assembly set on a magnetic plate at 20 RPM. Subsequently, 2 mL samples were withdrawn from the beaker at regular intervals of 15, 30, 60, 90, 120, 150, 180, 360, 720 and 1440 min. Volume of the buffer in the beaker was replaced by adding 2 mL of fresh buffer. The collected samples were quantified for the amount of released drug via UV plate reader at a wavelength of 254 nm. A standard curve of the ACV ranging between 0–100 µg was developed for this purpose.

### 2.9. Formulation of OC-PAP Based Nanoemulsion

Based on the drug release profile of the nanoemulsions, one formulation was selected with relatively sustained and complete release of the drug within the study period. A replica of this selected formulation (F10) was developed by replacing oleoyl chloride with the OC-PAP conjugate. The selected formulation with oleoyl chloride acted as the control for onward mucosal studies.

### 2.10. Characterization of the Selected Nanoemulsion

SEDDS formulation selected on the basis of drug release characteristics and its replica containing OC-PAP conjugate were diluted in distilled water at a concentration of 0.5% at room temperature. Droplet size and zeta potential characteristics for both of the nanoemulsions were established by dynamic light scattering utilizing a Malvern Zetasizer Nano ZSP (Malvern Panalytical Technologies, Malvern, UK) at 25 °C immediately after preparation [21].

### 2.11. Cytotoxicity Studies

The cytotoxicity potential of the selected nanoemulsions was evaluated via MTT (3-(4,5-dimethylthiazol-2-yl)-2,5-diphenyltetrazolium bromide) assay utilizing an HCT-116 cell line (a human colon cancer cell line) [22]. Briefly, HCT-116 cell suspension (density 1 × 10^5^ cells/mL) in 0.1 mL of minimum essential medium (MEM) was cultivated within a 96-well plate and incubated at 37 °C and 5% CO_2_. After 24 h, the cells were carefully washed with phosphate buffer saline (PBS) and 0.1 mL of nanoemulsions (0.5% *m*/*v* in MEM) of SEDDS formulation with OC (F7) and with OC-PAP (F10) were added, followed by incubation for 4 h at 37 °C and 5% CO_2_. Cells containing no formulation but MEM only and 1% solution of Triton X-100 in MEM were taken as negative and positive control, respectively. At the end of exposure period, the nanoemulsions were removed, washing with PBS was carried out twice and 0.1 mL of MTT solution (0.5% in MEM) was added. After another incubation of 4 h, the supernatant was removed and precipitates were dissolved in 0.1 mL of dimethyl sulfoxide (DMSO). Thereafter, the absorbance of DMSO solution was measured by a microplate reader at 570 nm with a background adjustment at 690 nm wavelength [23]. The percentage cell viability was calculated against the positive control as follows:

$$\mathrm{Cell}\;\mathrm{viability}\;\left[\%\right]=\frac{\mathrm{Absorbance}\;\mathrm{nanoemulsion}\;\mathrm{samples}}{\mathrm{Negative}\;\mathrm{control}}\times 100$$

### 2.12. Mucous Permeation Studies

The small intestine of the rabbit was cut along its length and placed over a smooth surface. The mucosal surface was gently rinsed with 0.1 mM phosphate buffer (pH 6.8) and then scrapped off from the underlying tissue with the help of a scraper. In order to purify, the collected rabbit mucus was mixed with 0.1 M NaCl solution, stirred for 1 h and afterward, the mucus/NaCl mixture was centrifuged at 13,000 RPM for 1 h. The supernatant containing the impurities and granular material was discarded and the purified form of the mucus was used for all the following experiments. Ethical approval for studies was obtained from Departmental Research Ethical Committee, Department of Pharmacy, COMSATS University Islamabad, Abbottabad Campus, Abbottabad, Pakistan [COM-SD-1610/PHM].

#### 2.12.1. Rotating Tube Method

The rotating silicon tubes method was followed to investigate the mucous penetration capability of the nanoemulsion [24]. Briefly, silicon tubes with 3 mm internal diameter were cut into small pieces of approximately 4–5 cm. The tube was filled with mucous obtained from rabbit intestinal mucosa, and one end of the tube was sealed with silicon cap. In the meantime, nanoemulsions were prepared by taking 50 mg each of drug-loaded SEDDS (F7 and F10) and adding separately to 500 µL of 100 mM phosphate buffer (pH 6.8). Afterward, 50 µL of each nanoemulsion was added to the open end of mucus-filled tubes in triplicate, sealed with silicon cap and placed under horizontal rotation for the study period of 4 h. At the end of the study period, the tubes were frozen overnight at −20 °C. Next morning, each tube was cut into smaller segments of 4 mm in length and was individually added to Eppendorf tubes followed by addition of 400 µL of phosphate buffer to each tube in order to extract the drug in mucus. The Eppendorf tubes were vortexed for 25–30 s and later, incubated for 30 min at temperature of 37 °C. Finally, 100 µL from each tube was added to 96-well UV plates to assess the samples photo-metrically at a wavelength of 254 nm. In total, 100 µL of phosphate buffer alone served as 0%, whereas 50 µg of drug dissolved in water and diluted with 400 µL of buffer acted as 100%. All the samples were added in duplicates to be assessed via spectroscopy.

#### 2.12.2. Trans-Well Plate Method

Mucosal permeation capability of the nanoemulsion was determined via a 24-transwell plate as described by Friedl et al. [25]. Firstly, the nanoemulsions were prepared by mixing 50 mg of SEDDS formulation (F7 and F10) with 2.5 mL of the 100 mM phosphate buffer (pH 6.8). In the meantime, a uniform barrier in the transwells was established by addition of 50 mg rabbit mucosa in each of the wells. Transwells (donor compartments) were filled with 500 µL of the nanoemulsions and placed in the recipient compartments filled with 1.3 mL of phosphate buffer (pH 6.8) in such a way that level of fluid in both compartments remained equal. A total of 300 µL of ACV solution (5 mg/mL) was added to 1.2 mL of phosphate buffer and 500 µL of this solution served as 100% and phosphate buffer (pH 6.8) alone acted as 0%. The plate was wrapped in an aluminum foil and placed on a rotatory surface set at a speed of 50 RPM in an incubator at 37 °C. A total of 100 µL of the samples were collected from the receiver compartment at time points of 30, 60, 90, 120, 150 and 180 min. Volume was maintained in the recipient compartment after withdrawal of each sample by adding 100 µL of fresh phosphate buffer (pH 6.8) maintained at 37 °C. The collected samples were quantified photo-metrically via Microplate reader at the wavelength of 254 nm. The amount of the ACV permeation was calculated in reference to permeation of the 100% and 0% references.

#### 2.12.3. Determination of Mucosal Permeation

**Figure 2:**
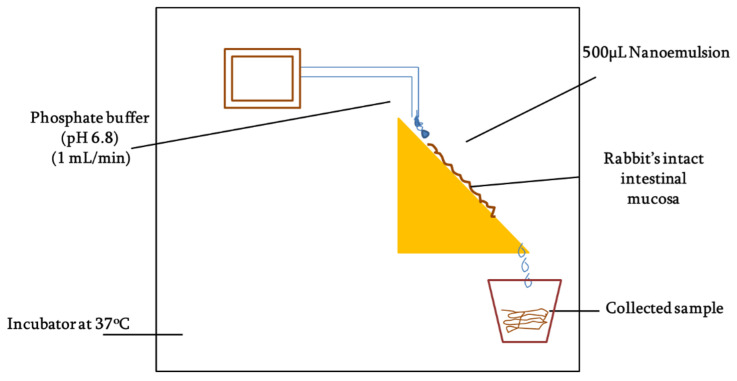
Pictorial description of the setup for mucosal permeation study.

Mucous penetration potential was determined using intact rabbit intestinal mucosa following the method as described by Griessinger and co-authors with slight modification [26] as illustrated in Figure 2. Briefly, rabbit intestine was cleaned with normal saline and then cut into smaller fragments of 2 × 3 cm. The intestinal segments were fixed to vertically half-cut 50 mL falcon tubes and rinsed with phosphate buffer (pH 6.8). The tubes were placed on an inclined surface that could provide 45° angle and system was placed in incubator maintained at 37 °C. In total, 50 mg of each SEDDS formulation (F7 and F10) was added to 2 mL 100 mM phosphate buffer (pH 6.8) to form nanoemulsions. Afterward, 500 µL of the nanoemulsion was applied to intestinal segments and allowed to penetrate for 5 min followed by a continuous flow of 100 mM phosphate buffer (pH 6.8) over the mucosa at a rate of 1 mL/min. The free-flowing rinsing buffer was collected all along the study into the recipient containers changing every 30 mins up to a time period of 3 h. The collected samples were photo-metrically assessed at the wavelength of 254 nm. The amount of the ACV permeation was calculated in comparison to the 100% and 0% reference standards.

### 2.13. Statistical Data Analysis

All the results are stated as means (±SD) of at least three experiments (triplicate samples). Data were analyzed utilizing the appropriate statistical tools such as Student’s *t*-test, two tails with 95% confident interval (*p*-value < 0.05) as the minimal level of significance.

## 3. Results

### 3.1. Synthesis and Characterization of OC-PAP

Conjugation occurred through the formation of an amide bond between the carbonyl group of the oleoyl chloride and the amino group of the papain resulting in the formation of the OC-PAP conjugate through condensation. The conjugate appeared as a pale yellowish liquid with a characteristic order of papain.

A Bradford assay was performed for the quantitative determination of the papain immobilization on the oil. Absorbance values obtained for the standard curve from UV spectroscopy were plotted on a graph with a concentration on the x-axis and absorbance on the y-axis. The assay demonstrated the papain content to be 45 ± 8.28 µg/mg of conjugate oil. Wavenumbers (cm^−1^).

**Figure 3:**
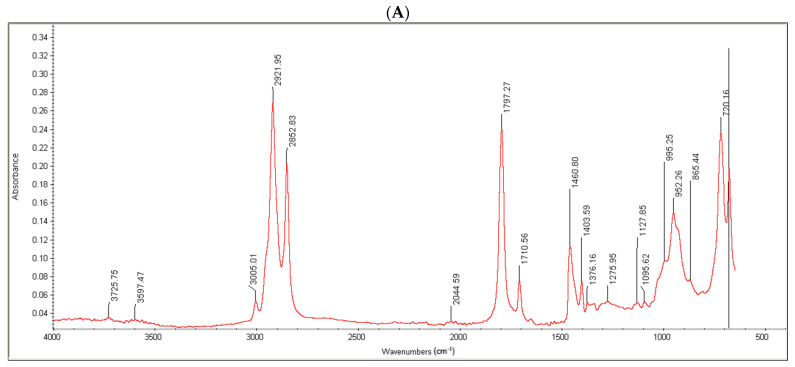

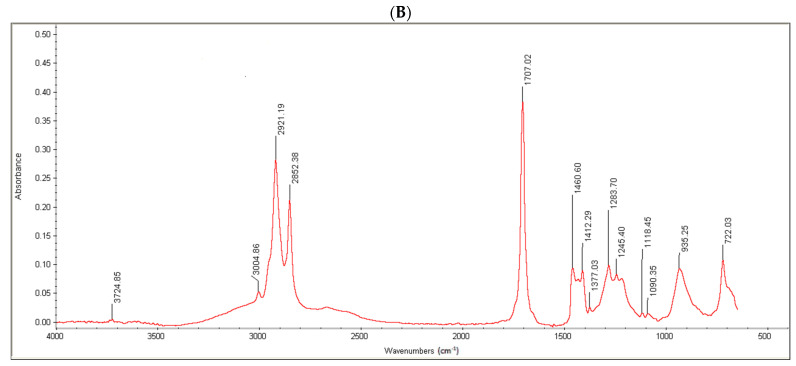
FTIR spectra of Oleoyl chloride (**A**) and OC-PAP conjugate (**B**).

Moreover, the conjugate formation was confirmed via FTIR analysis. Oleoyl chloride and OC-PAP were evaluated via FTIR and the resultant spectra are shown in Figure 3. The FTIR spectra of oleoyl chloride and OC-PAP conjugate show distinctive features for the formation of the amide bond, such as a shift in the peak of C=O at 1797/cm in the case of oleoyl chloride to 1707/cm for the newly developed OC-PAP conjugate and the N–H stretching in the range of 3400–3000/cm. 

**Figure 4:**
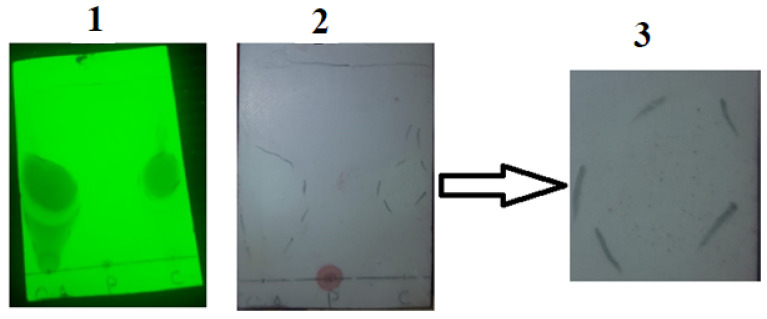
Thin layer chromatography of oleoyl chloride (O), papain (P) and oleoyl chloride-papain conjugate (C). The figure is an illustration view under ultraviolet light (1), a view with the naked eye (2) and a zoom-in of the potential area representing violet spots of immobilized papain (3).

Another way to qualitatively assure the conjugate formation was thin layer chromatography. Sample spots consisted of a drop of oleoyl chloride, papain and the oleoyl chloride-papain conjugate. As shown in Figure 4, “1” shows the appearance of the TLC plate when illuminated under a short wavelength UV lamp. This helped to mark the flow of oils along the length of the TLC plate within the mobile phase. The middle part of Figure 4, i.e., “2”, highlights the separation achieved after spraying the plate with a ninhydrin solution. The very left spot was of oleoyl chloride that served as the control. In the center, the papain solution “P” was spotted at the point of the application of the drop that appeared as a dark bluish-purple spot after the application of a marker, i.e., ninhydrin solution. This meant no affinity of the papain within the mobile phase. The third sample spot labeled as C on the TLC plate consisted of the OC-PAP conjugate. In “B”, very small purple-colored dots (the same as with papain) can also be seen within the encircled area for the conjugate, confirming the formation of the OC-PAP conjugate. Picture “3” is the magnified image of the outlined area from “2” marking the trajectory traveled by the conjugate in the mobile phase. The movement of papain along with the oil confirms the conjugate formation.

### 3.2. Selection of Formulation

**Figure 5:**
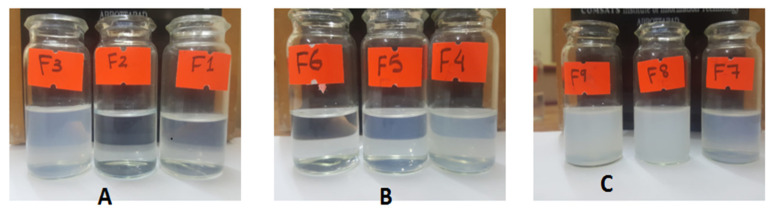
Set of nanoemulsions developed with Glycerin (**A**), Polyethylene glycol (**B**) and Propylene glycol (**C**) as solvents.

Out of nine formulations, three formulations (one each with a different co-solvent) were selected on the basis of ease of dispersibility and similarity with respect to the slight milky appearance that is indicative of the globule size. Upon the addition of 25 mg of SEDDS based on glycerin F1, F2, and F3 separately in a 10 mL buffer, the emulsions formed demonstrated a varying degree of dispersibility and transparency with F3 being reasonably milky to assure globule formation with slight shaking, followed by F1 showing moderate turbidity and F2, which was an almost clear solution as described in Figure 5A.

As displayed in Figure 5B, nanoemulsions formed with the same ratios as above from the SEDDS concentrate based on polyethylene glycol as co-solvent F4, F5 and F6 exhibited a higher degree of similarity in their ease of dispersibility. F4 and F5 nanoemulsions were easily dispersible and were close to each other in terms of transparency. However, F6 was comparatively clearer than the other two emulsions. F4 was picked based on its similarity to the chosen formulation from other groups and of course its moderation in dispersibility and transparency.

**Figure 6:**
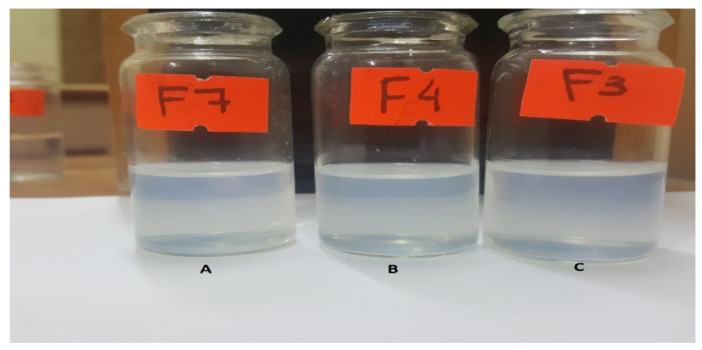
The selected formulations F7 (**A**), F4 (**B**) and F3 (**C**), were relevant in their dispersibility potential and transparency of the final emulsion as evident here.

Formulations F7, F8 and F9 were a little hard to disperse compared to the formulations belonging to the previous two groups and, in general, the resultant emulsions shifted towards a higher degree of turbidity. F8 and F9 exhibited poor dispersibility and were translucent in appearance. F7 illustrated comparatively better ease of dispersibility and turned out to be comparable to the two picked formulations from previous groups in terms of appearance, as depicted in Figure 5C. All the selected formulations belonged to three different classes, as illustrated in Figure 6, and they look similar to the naked eye.

### 3.3. Hydrophobic Ion Pairing

In the line of SEDDS, hydrophobic ion pairing has proved to be a time-tested strategy to incorporate hydrophilic drugs, therefore, it was applied for ACV. The results demonstrated 89.4%, 96.1% and 98.8% of the ion-pairing efficiency with the molar ratios of 1:2, 1:3 and 1:4, respectively.

### 3.4. Drug Release Profile

**Figure 7:**
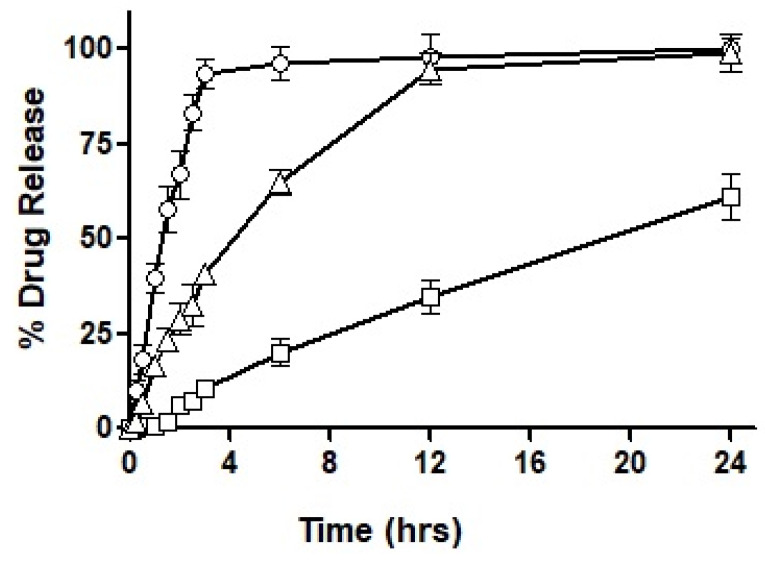
Percentage of ACV release from the nanoemulsions prepared from F3 (◯), F4 (□) and F7 (△) separately in 0.1 M phosphate buffer pH 6.8 for 24 h using the dialysis tube method.

After loading the hydro-phobically ion-paired drug into the selected formulations, i.e., F3, F4 and F7, the drug release profile was obtained over an extended period of 24 h. The results acquired for the drug release profile are demonstrated in Figure 7.

As seen in the figure, F3 showed immediate drug release with the first burst of drug release starting at 10 min. Almost 95% of the total drug was released completely within the first three hours of the experimental study. On the other hand, F4 exhibited very negligible drug release during the first hour, by the end of the third hour 10% of the drug was released and as the study progressed to 6 h only 20% of the drug had been released. A maximum drug release observed after a period of 24 h was 61%, therefore, demonstrating an incomplete drug release within the study time. In the next, F7 exhibited a sustained release trend extended over a period of 24 h, with approximately 28% of drug release during the first two hours. Afterward, 45% of the drug release was observed in the next 4 h which is considered the most crucial time for absorption. In the following 6 h, another 20% of the drug was released to the surrounding media leading to over 90% of drug release during the 12 h of time. Both F3 and F7 illustrated almost complete release of the drug over a period of 24 h.

**Figure 8:**
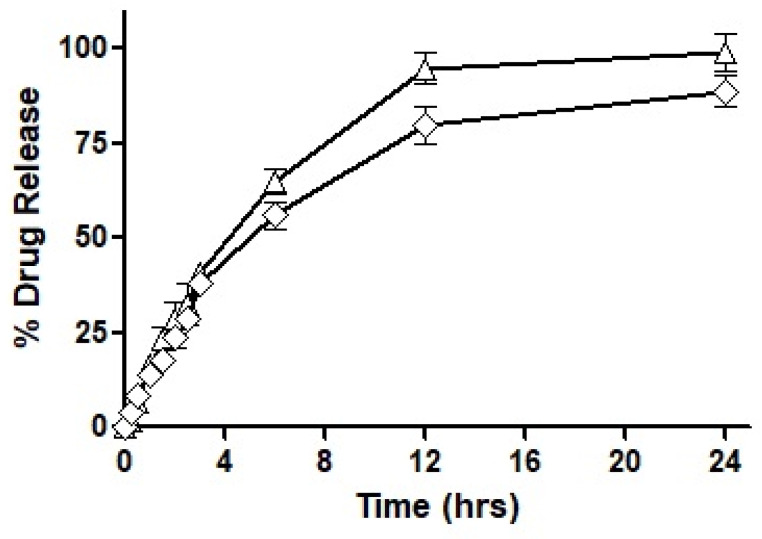
Comparison of the Percentage of ACV release from the nanoemulsions prepared from F7 (△) and F10 (◇) using dialysis tube method.

Based on the outcomes of the study, it was concluded that F7 had the most suited drug release profile being sustained as well as complete, and therefore, it was chosen for our mucosal permeation studies. However, this formulation had to act as a ‘baseline’ and the new formulation was fabricated as a replica of F7. This new formulation named F10 was prepared by replacing oleoyl chloride with the OC-PAP conjugate in order to increase the permeation across mucus and the rest of the formulation, process and drug loading remained unaffected. In order to assure that the change in the formulation has no significant impact on the release of the drug, a drug release profile for F10 was also constructed and is shown in Figure 8. The drug release from F7 and F10 had a very similar pattern up to 3 h with only a slight difference. Afterward, the release of the drug was more sustained with a 56% drug release observed up to 6 h and at the end of the study, approximately 90% of the total drug had been released from F10. Statistically, the release profile of F10 was insignificantly different than F7.

### 3.5. Characterization of the Nanoemulsion

The selected SEDDS formulation and its OC-PAP-containing replica were primarily characterized with respect to average droplet size, the size distribution and zeta potential values and the details are described in Table 2. The average diameter of droplets did change significantly when we compare formulations F7 and F10; moreover, a deeper look at the size distribution illustrates the difference in behavior. As depicted in Supplementary Figure S1A, the F7 formulation shows two major peak areas; the dominant peak of 14.7 ± 2.97 nm representing approximately 75% of the particles and a secondary peak at 407.1 ± 85.52 nm. In contrast, F10 is characterized by a dominant peak at 315.7 ± 156.3 nm representing 97% of the nanoemulsion droplets. Zeta potential values for all nanoemulsions remained neutral to slightly negative. The growth in particle size or flocculation may be attributed to the changes in zeta potential after the addition of OC-PAP in F10, i.e., moving from negative zeta potential values to almost neutral values.

### 3.6. Cytotoxicity Analysis

**Figure 9:**
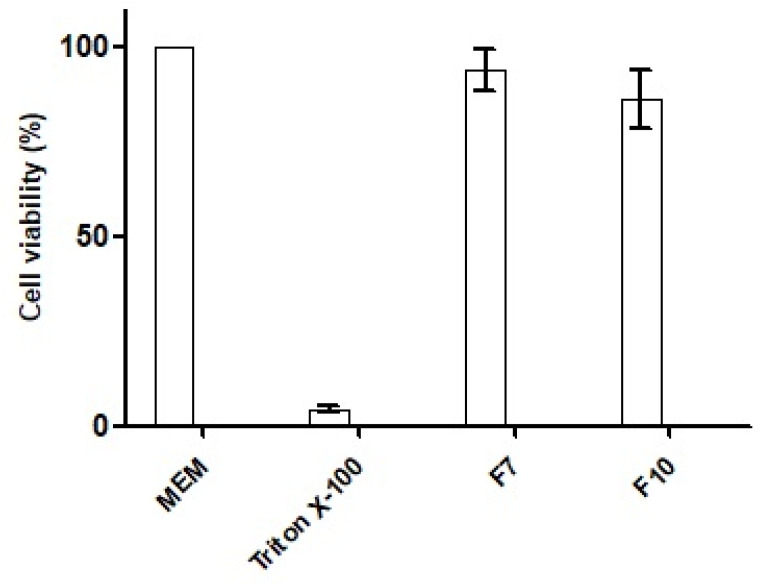
Viability percentage of HCT-116 cells after 4 h of exposure to 0.5% nanoemulsions with OC (F7) and OC-PAP (F10). MEM alone served as negative control, whereas, 1% triton X-100 solution in MEM was taken as positive control. The values are described as means of three experiments ± standard deviation.

The cytotoxic potential of the nanoemulsions was investigated by measuring the percentage of cell viability in the MTT assay. The outcomes of the experiment after exposure to HCT-116 cells with the SEDDS formulation with OC (F7), OC-PAP (F10), and negative and positive control are depicted in Figure 9. Both of the nanoemulsions demonstrated cell viability of more than 80% after an incubation of 4 h and therefore, can be considered safe.

### 3.7. Mucous Permeation Potential

#### 3.7.1. Trans-Well Method

**Figure 10:**
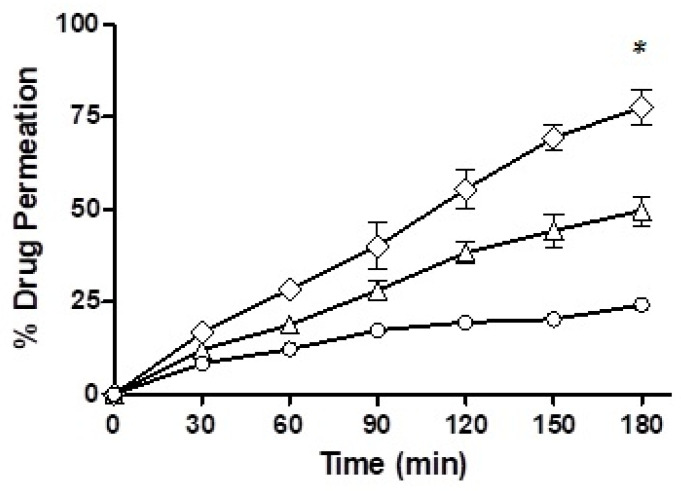
Percentage of ACV permeated through a layer of rabbit intestinal mucus via free drug solution (◯), loaded into F7 (△) and F10 (◇) studied over a period of 3 h. * indicates that the outcomes of F10 are significantly different than F7.

Mucus permeation potential was determined for the drug-loaded F7 and F10 via the trans-well method and the results are depicted in Figure 10. In the starting half an hour of the study, the drug penetration from the nanoemulsions was very comparable with F7, which showed a mucous permeability of 12% while there was a 16.69% mucosal drug permeation from F10. However, towards the end of the study, after a time-lapse of 3 h, F7 showed a mucous permeation potential of 49.70%, F10 demonstrated 77.67% and the drug free from solution only 24.19%. Of all the three formulations, F10 showed the strongest mucous permeation capability owing to the presence of immobilized papain and statistically, the result was significantly different than F7.

#### 3.7.2. Rotating Silicon Tube Method

**Figure 11:**
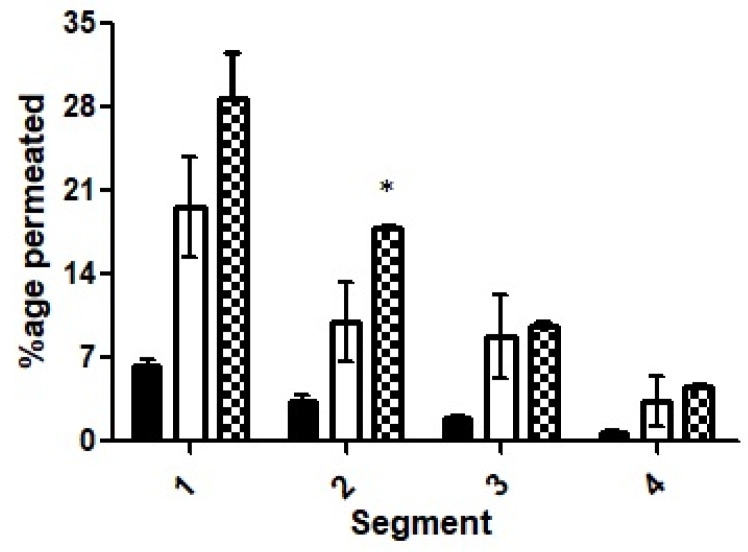
Penetration capacity of ACV while an aqueous solution (black bars), loaded into selected formulation F7 (white bars) and loaded into OC-PAP formulation (dotted bars) analyzed via rotating silicon tube method for 4 h. * indicates that the outcomes of F10 are significantly different than F7.

The results obtained for the rotating silicon tube method are illustrated in Figure 11. The segments were evaluated on the basis of their proximity to the added emulsion. The first segment for each of the three formulations (F7, F10 and the control) that was nearest to the formulations showed the highest penetration. The first segments of the silicon tubes demonstrated about five times more penetration for F10 as compared to the ACV solution and 1.5-times more compared to selected formulation F7, however, this was statistically insignificant.

A significant difference existed only for segment two while making a comparison between the nanoemulsions F7 and F10. Moreover, the mean values for F7 or F10 were significantly different when compared to the ACV solution. Moreover, a shift from a large mean difference of the formulations of the first segments to a smaller value of mean difference for the second, third and fourth segments for F10 suggests efficient and consistent permeation. All in all, cumulatively, more than 65% of the ACV penetrated to various levels from the surface in the targeted F10 formulation as compared to less than 15% for the control and around 40% for the nanoemulsion of the selected formulation.

#### 3.7.3. Mucous Penetration Potential

**Figure 12:**
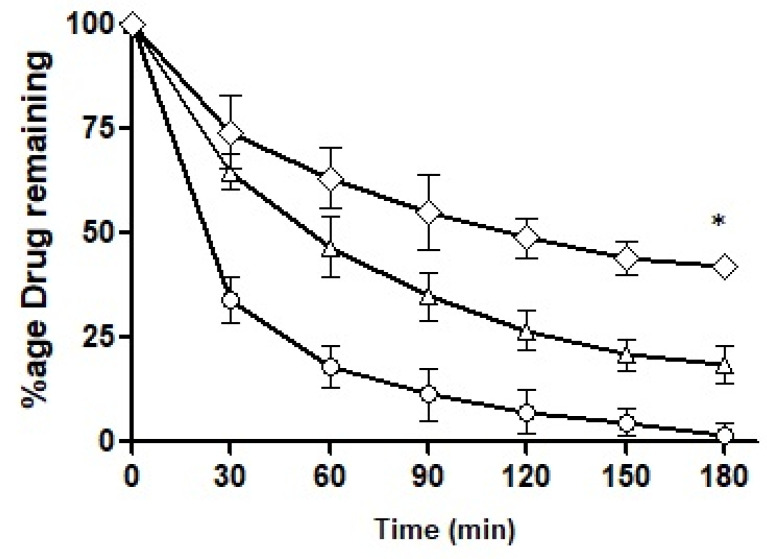
Percentage of ACV remaining on excised rabbit intestinal mucosa as free drug solution (◯), loaded into F7 (△) and F10 (◇) being continuously rinsed with 0.1 M phosphate buffer pH 6.8 for 3 h. * indicates that the outcomes of F10 are significantly different than F7.

The intestinal retention of ACV as a free solution and loaded into the formulations was explored and the results obtained are illustrated in Figure 12. By the end of 3 h, F10 retained up to 50%, F7 up to 22% and the control less than 3%. F7 showed a somewhat sharp fall in its intestinal retention potential and the control exhibited a drastic fall as time passed. F10 was found to be superior in its intestinal retention ability and displayed significantly different penetration potential as compared to F7. The mucolytic papain cleared the pathway of entry of the nanoemulsions in the mucosal structure. Particles with deeper penetration tend to stay for a longer time duration exhibiting longer intestinal retention. F10 had mucolytic activity thus showing the prolonged retention capability while on the other hand, the control had no such mucous permeating system so it lacked the ability to be retained inside the mucosal structure leading to a poor retention time of the drug particles. Almost all the drug had washed out by the end of 3 h.

## 4. Discussion

In the current study, an attempt was made to enhance the oral bioavailability of a very potent antiviral drug ACV, belonging to BCS class III, i.e., exhibiting good (pH-dependent) aqueous solubility but low permeation capability [27]. To achieve our goal of enhancing the mucosal permeation of the drug, one of the re-emerging trends in the field of drug delivery, i.e., utilization of SEDDS (a commonly known carrier for lipophilic drugs) was employed [28,29,30]. SEDDS may follow variable mechanisms to increase bioavailability but before being in contact with the gastrointestinal fluids, SEDDS form oil in water emulsion as a result of the agitation produced naturally due to the peristaltic movement of the gastrointestinal system [31]. The selection of the ingredients for the formulations was based upon the previous studies regarding SEDDS [20,32,33], with some modifications, in particular the co-solvent. The oil used in the SEDDS keeps the drug in a solubilized state until the drug is released at the absorption lining and an emulsified system largely increases the surface area optimizing the partitioning of the drug between the two phases, i.e., oil phase and aqueous phase, leading to a drastic increase in drug solubility and absorption.

On one hand, the incorporation of a hydrophilic drug into the SEDDS was achieved via ion pairing with a hydrophobic moiety and on the other hand, an enhancement of mucus penetration of SEDDS was targeted by immobilization of proteolytic enzyme on its surface. An increasing concentration of sodium deoxy-cholate, the counter ion, was evaluated as the ion pairing is directly proportional to the concentration of the surfactant (until the micelles start to develop) [33]. In the literature, the effect of pH at which ion pairing takes place is discussed in detail, but in the case of ACV, the pH-dependent solubility did not provide flexibility.

Proteolytic enzyme immobilization was accomplished during the course of the study via synthesis of the papain-functionalized excipient OC-PAP, though the activity of the enzyme after immobilization might have slightly decreased. Papain is one of the prime components in the list of proteases that was covalently linked with oleoyl chloride (a halide derivative of oleic acid) via the amide bond. From a chemistry point of view, conjugation of acyl halides to amines is considered a very vigorous process while aqueous conditions are maintained [15]. The extent of immobilization of papain in the OC-PAP conjugate was assessed via the Bradford assay. The papain content was found to be in a reasonable range of 45 µg/mg, representing a reaction efficiency of around 90%. The unreacted papain contents may have remained within the excess water that was added at the end of the conjugation reaction. Bradford assay described the immobilization of papain quantitatively while FTIR analysis and TLC were performed to qualitatively confirm the conjugate formation. FTIR spectra confirmed the formation of the conjugate with distinctive changes such as stretching in the N–H bond and shift of the C=O due to the amide bond formation [34,35]. Thin layer chromatography was carried out to investigate the formation of the conjugate by using a mixture of hexane, ethyl acetate and acetic acid as the mobile phase. The selected combination for the mobile phase was based on the idea that it should have sufficient affinity for the oil but act as a non-solvent for the papain. Therefore, as the mobile phase traveled through the TLC plate taking the oil components from the area of application and also causing elution, in the meantime, the papain remained unaffected. After treatment with ninhydrin solution, the developed TLC plate showed the appearance of bluish-purple colored spots at the area of application of the papain and for the OC-PAP conjugate in the marked area for oleoyl chloride. The appearance of the same colored spots at two positions signifies the immobilization of papain in the oleoyl chloride forming an OC-PAP conjugate. This depicts the occurrence of a condensation reaction and amide bond formation between the papain and the oleoyl chloride. The protease-functionalized conjugate and the resultant nanoemulsions were evaluated for characteristic features and improved drug permeation across the rabbit intestinal mucosa, respectively.

The drug release profile was used for the selection of an optimized formulation for the hydrophobic ion-paired drug. The use of different concentrations of surfactant, co-surfactant and, in particular, varying the co-solvent had a significant impact on the drug release; from an immediate release with glycerin to an incomplete release even in 24 h with polyethylene glycol. However, propylene glycol as a co-solvent was considered ideal for providing a sustained and substantial amount of drug release within the study time. Importantly, replacing the oleoyl chloride with OC-PAP had a statistically insignificant effect on the release of the drug pattern, the slight reduction in the extent of release during the study period with the modified conjugate may be attributed to the increased particle size of the droplets and therefore, less surface area available for drug release [36].

SEDDS based on a modified excipient illustrated a consistent enhancement in permeation when evaluated in vitro with various mucus-based studies. Investigation of the penetration potential of the nanoemulsions was carried out by performing three studies on the rabbit intestinal mucosa in order to simulate the external conditions with the natural physiology. The study’s outcome ensured the enhanced mucosal permeability of the nanoemulsions. This could be justified based on the fact that 5% of the mucosal structure consisting of the mucin and papain, owing to its potent mucolytic activity, cause lysis of the peptide linkages of mucin thereby leading to increased transportation of the ACV into the mucosal barrier. The rotating silicon tube method was adopted to determine the penetration capability of the SEDDS and it was carried out to mimic the external conditions with the peristaltic movements of the body in order to evaluate the penetration potential of the SEDDS in a normal physiological environment. The study results indicated an elevated penetration strength of the SEDDS that was comparable to the penetration ability reported in previous studies [37,38]. The trans-well method was performed to study the migratory property of the SEDDS through the mucosal barrier at a temperature of 37 °C to simulate internal conditions. The efficiency of the SEDDS was investigated by comparing the permeation potential of the developed SEDDS with the pure drug that was free of mucolytic enzyme immobilized in it. The outcome of the experiment highlighted the enhanced permeation of the SEDDS across the rabbit intestinal mucosa by the proteolytic activity of the papain conforming to the data reported by Muller and co-workers [39]. An experiment was set up to explore the intestinal retention of all three formulations. The developed SEDD formulation with mucolytic activity exhibited a higher retaining potential compared to other formulations and the control. The mucolytic activity of the papain helped to migrate the nanoemulsions deeper into the mucosal layer where they were found to be retained for a longer time, as suggested by their prolonged retention times.

The idea of combining the ion-pairing technique with SEDDS and the proteolytic enzyme immobilization is very promising for the model drug, ACV. It can further be extended to other drugs with low oral bioavailability, however, a series of detailed in vivo studies must be designed and conducted to confirm the outcomes achieved from in vitro data.

## 5. Conclusions

The strategy of papain immobilization onto SEDDS and enhancing the mucosal permeation of ACV-loaded nanoemulsions is a first step in improving oral bioavailability. The idea of incorporating the drug as a hydro-phobically ion-paired system proved to be of significant importance and the drug release profile revealed fundamental characteristics from the optimized formulation. Immobilization of papain not only improved the mucosal permeation, but the SEDDS were found to have an increased retention time due to deeper permeation of the particle in the protein structures of the mucous. Therefore, it can be safely concluded that SEDDS are a promising approach for delivery of even BCS class III drugs and its combination with mucolytic enzymes can be implied successfully to overcome the issues of drug permeation, absorption and bioavailability.

## Figures and Tables

**Table 1:** Composition of numerous SEDDS formulations. The quantities are expressed in milligrams (mg).

Formulation	Oleoyl Chloride	Kolliphore	Transcutol	Glycerin	Polyethylene Glycol 400	Propylene Glycol
F1	100	400	300	200	-	-
F2	100	300	400	200	-	-
F3	100	350	350	200	-	-
F4	100	400	300	-	200	-
F5	100	300	400	-	200	-
F6	100	350	350	-	200	-
F7	100	400	300	-	-	200
F8	100	300	400	-	-	200
F9	100	350	350	-	-	200

**Table 2:** Characterization of nanoemulsions.

SEDDS Formulation	Nanoemulsion Average Size (nm)	Polydispersibility Index	Zeta Potential
F7	116.3	0.357	−7.73
F10	334.5	0.481	−0.601

## Supplementary Materials

The following supporting information can be downloaded at: https://www.mdpi.com/article/10.3390/pharmaceutics14081584/s1, Figure S1: The size distribution of SEDDS formulation F7 [A] and F10 [B] measured after getting diluted in distilled water.
