# Supplementary material for: Papain-Decorated Mucopenetrating SEDDS: A Tentative Approach to Combat Absorption Issues of Acyclovir via the Oral Route
Source: Pharmaceutics. 2022 Jul 29;14(8):1584. doi: 10.3390/pharmaceutics14081584 (PMC9412565; doi:10.3390/pharmaceutics14081584)
Supplement: Supplementary file 1 [file pharmaceutics-14-01584-s001.zip › pharmaceutics-1800423-supplementary.pdf]

Supplementary Materials: Papain-decorated Mucopenetrating SEDDS: A tentative approach to combat absorption issues of Acyclovir via the oral route

Arshad Mahmood, Rabbia Haneef, Ahmad Z. Al Meslamani, Mohammad F. Bostanudin, Muhammad Sohail, Muhammad Sarfraz, Mosab Arafat

[A]

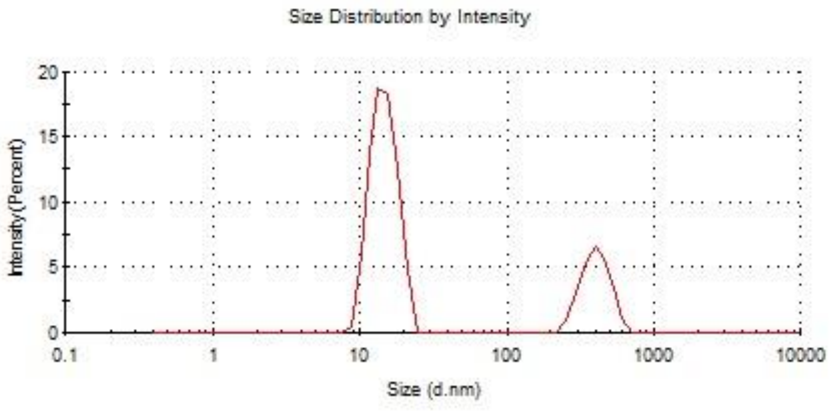

[B]

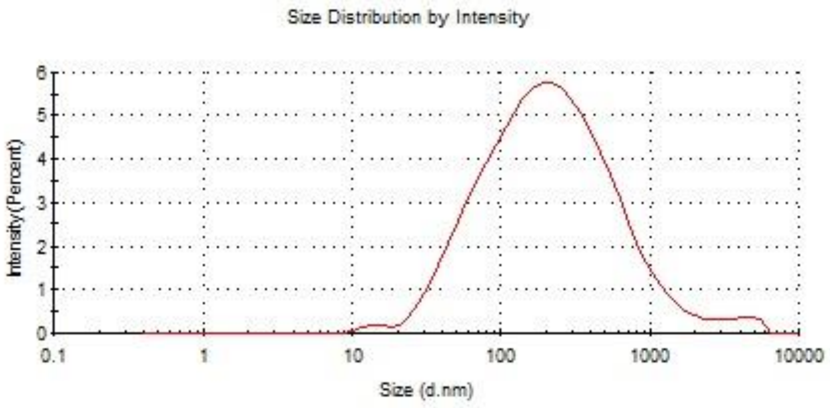

Figure S1. The size distribution of SEDDS formulation F7 [A] and F10 [B] measured after getting diluted in distilled water.
